# A Scalable AI-Based System for Evaluating and Enhancing Responsible Media Coverage of Suicide: A Multi-Site, Multi-Language Implementation Study

**DOI:** 10.1192/j.eurpsy.2026.10215

**Published:** 2026-07-31

**Authors:** Y. Levi-Belz, Z. Elyoseph

**Affiliations:** University of Haifa, Haifa, Israel

## Abstract

**Introduction:**

Irresponsible media coverage of suicide can increase suicide rates through the “Werther effect,” while responsible reporting can reduce risk and stigma. Although the WHO has issued clear guidelines for responsible reporting, adherence remains inconsistent worldwide due to structural barriers and the lack of real-time tools for journalists.

**Objectives:**

This study aimed to develop and rigorously evaluate a multilingual GenAI-based system that can automatically assess and enhance suicide-related news articles to align with WHO guidelines, offering a scalable pathway for more responsible coverage.

**Methods:**

A total of 120 suicide-related articles (English, Hebrew, and French) were systematically selected and independently evaluated for guideline adherence by both expert human raters and the AI system. The articles were automatically revised using the AI correction module. Post-enhancement, all articles were re-evaluated by the system and by a new set of human raters to compare changes in adherence levels.

**Results:**

The AI system demonstrated high agreement with human raters at baseline (ICC = .77), with slight conservative bias in some languages. Following the AI-based enhancement bot, both AI and human evaluations confirmed significant improvements in guideline adherence (mean scores increased from ~45% to ~85%), with no significant post-enhancement differences between human and AI ratings.

**Image 1:**
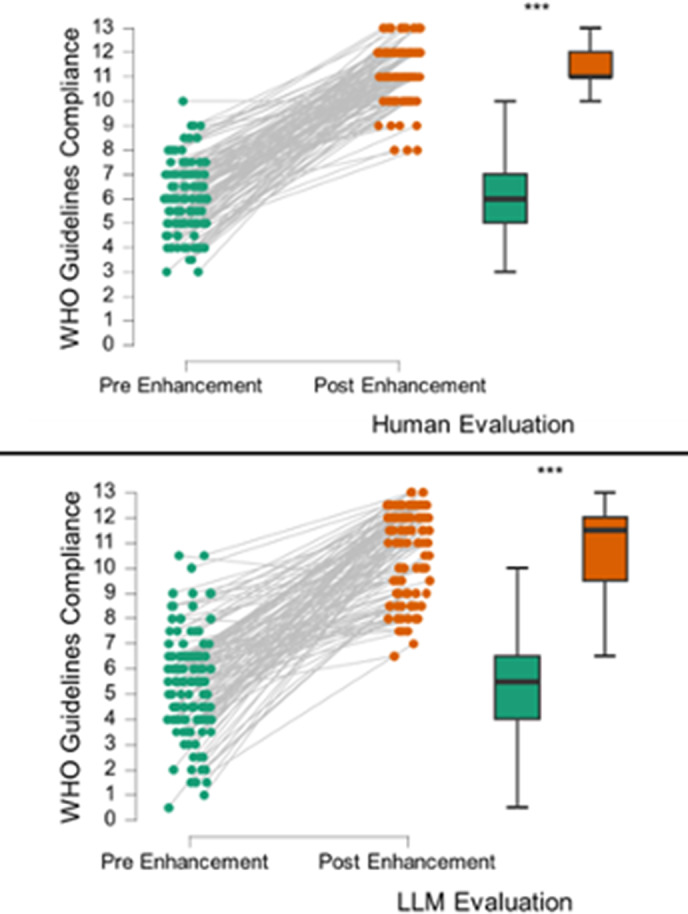
Image 1: Long description.

**Conclusions:**

This study provides initial empirical support for using GenAI to automatically evaluate and enhance suicide-related media coverage across languages and contexts. By offering journalists a practical, real-time tool for producing accurate, ethically sound content, this approach can help mitigate suicide contagion, increase public awareness, and foster a global metric for responsible reporting.

**Disclosure of Interest:**

None Declared

## Long descriptions

### Image 1: Long description

The figure consists of two vertically stacked panels. Both panels share a Y-axis labeled W H O Guidelines Compliance with a scale from 0 to 13. The X-axis categories are Pre Enhancement and Post Enhancement.

Top Panel: Human Evaluation.

- Pre Enhancement: A green dot plot shows scores clustered between 3 and 10. A corresponding green box plot to the right shows a median of 6, with an interquartile range from 5 to 7.

- Post Enhancement: An orange dot plot shows scores clustered between 8 and 13. Gray lines connect individual data points from Pre to Post, showing a consistent upward trend. A corresponding orange box plot shows a median of 11.5, with an interquartile range from 11 to 12.

- Three asterisks above the box plots indicate statistical significance.

Bottom Panel: L L M Evaluation.

- Pre Enhancement: A green dot plot shows scores ranging from 0.5 to 10.5. The green box plot shows a median of 5.5, with an interquartile range from 4 to 6.5.

- Post Enhancement: An orange dot plot shows scores ranging from 6.5 to 13. Gray lines show a steep upward trend for most individual data points. The orange box plot shows a median of 11.5, with an interquartile range from 9.5 to 12.

- Three asterisks above the box plots indicate statistical significance.

Navigate back to Image 1:.

